# Sex differences in coronary heart disease and stroke mortality: a global assessment of the effect of ageing between 1980 and 2010

**DOI:** 10.1136/bmjgh-2017-000298

**Published:** 2017-03-27

**Authors:** Sophie H Bots, Sanne A E Peters, Mark Woodward

**Affiliations:** 1 The George Institute for Global Health, University of Oxford, Oxford, UK; 2 The George Institute for Global Health, University of Sydney, Sydney, New South Wales, Australia; 3 Department of Epidemiology, Johns Hopkins University, Baltimore, Maryland, USA

## Abstract

**Background:**

Cardiovascular disease mortality rates are well known to be lower in women than men and to increase with age. Whether these sex and age effects have changed over recent decades, and how much they differ by country, is unclear.

**Method:**

From the WHO Mortality Database, we obtained age-specific and sex-specific coronary heart disease (CHD) and stroke mortality rates for the world's most populous countries with data available between 1980 and 2010. We calculated age-specific, country-specific and period-specific men-to-women CHD and stroke mortality rate ratios for 26 countries and compared the differences between and within countries over time.

**Results:**

CHD and stroke mortality decreased substantially between 1980 and 2010 in most countries, in both sexes. Mostly there was an attenuation of the effect of ageing over calendar time, more so in men than in women. CHD mortality was higher in men than in women throughout adulthood, but the magnitude of the difference varied by age. Men-to-women CHD mortality rate ratios were 4–5 in middle age (30–64 years) and 2 thereafter (65–89 years). Stroke mortality was more similar between sexes, with men-to-women stroke mortality rate ratios of around 1.5–2 until old age.

**Conclusions:**

While CHD and stroke mortality rates declined considerably between 1980 and 2010 in both sexes, there was some indication for stronger age-specific reductions in CHD in men than women. Mortality from CHD and stroke remains higher among men than women until old age across a range of economically, socially and culturally diverse countries.

**Key questions**

**What is already known about this topic?**

- Cardiovascular disease (CVD) is the leading cause of death worldwide, yet important differences exist between men and women.
- Men generally develop CVD at a younger age and have a higher risk of coronary heart disease (CHD) than women. Women, in contrast, are at a higher risk of stroke, which often occurs at older age.

**What are the new findings?**

- CHD and stroke mortality rates have declined over the past decades in both sexes.
- Mortality from CHD and stroke remain higher among men than women until old age.
- Age-specific reductions in CHD rates may have been stronger in men than women.

**Recommendations for policy**

- A better understanding of sex differences in CVD is needed to prevent and treat CVD more efficiently in women and men.
- Continued efforts are needed to debunk the notion that CVD is a man's disease.

## Introduction

Cardiovascular disease (CVD) is still the leading cause of death worldwide. In 2013, 32% of deaths in men and 35% of deaths in women were due to CVD.^1^ ^2^ Despite the higher percentage of CVD deaths in women, CVD is still widely considered as a man's disease. This assumption stems from the historical misperception that the manifestation of CVD among women is uncommon or is not characterised by the same symptoms as it is in men.^3^ ^4^

Men generally develop CVD at a younger age and have a higher propensity of developing coronary heart disease (CHD) than women. Women, in contrast, are at a higher risk of stroke, which often occurs at older age.^5^ ^6^ Hence, stroke and CHD mortality rates in middle-aged men are typically higher than in middle-aged women, a difference that may persist throughout most of the lifetime.^7^ ^8^ Successes in primary and secondary strategies have led to major reductions in age-standardised CVD mortality rates, especially in Western countries where case fatality rates have dropped significantly and where access to prevention and healthcare is typically higher than in other parts of the world. At present, however, it remains unknown to what extent the reductions in age-standardised CVD mortality rates have affected the sex difference in CHD and stroke mortality rates, standardised by age and across the life course.^9^ Nor has there been a comprehensive assessment of as to what extent such changes in sex differences differ across geographically diverse regions.

It is evident that older people are at a higher risk of dying from CHD and stroke than younger people. Often researchers opine that women ‘catch up’ with men after the menopause, but whether this is true in this context is debated.^10^ Furthermore, it is unknown whether the effect of ageing, in both sexes, is the same now as it was 20–30 years ago, while a comprehensive analysis of the effects of ageing on the relative chances of dying from CHD and stroke is lacking.

In the present study, we therefore conducted a global evaluation of trends in sex and age differences in CHD and stroke mortality rates between countries and over recent times.

## Methods

Data were used from the WHO Mortality Database.^11^ This compiles deaths registered in national vital registration systems, with underlying cause of death as coded by the relevant national authority. The database contains the number of deaths by country, year, sex, age group and cause of death, and population size by country, year, sex and age group. To obtain a diverse and representative sample of the world's population, we extracted data for the 50 most populous countries worldwide. From these, we selected those countries for which population size and mortality data were available for 1980, 1990, 2000 and/or 2010, or for up to four adjacent years. A total of 26 countries were thus included: Argentina, Brazil, Canada, China, Colombia, Egypt, France, Germany, Italy, Japan, Malaysia, Mexico, Peru, the Philippines, Poland, Russia, South Africa, South Korea, Spain, Thailand, Turkey, UK, Ukraine, USA, Uzbekistan and Venezuela (see online supplementary eTable 1).

10.1136/bmjgh-2017-000298.supp1 supplementary eTables

### End points

The primary end points were mortality from CHD and stroke, as defined by the International Classification of Disease (ICD). With some exceptions (see online supplementary eTable 2), countries coded mortality using the 9th edition of ICD until 2000 and using the 10th edition from 2000 onwards.^11^ ICD codes for CHD and stroke, respectively, were B27 and B29 in ICD-9 and were I20–I25 and I60-I69 in ICD-10. The estimated net change in cause-specific mortality rates, due to changes in classification rules, from ICD-9 to ICD-10 has been estimated to be a 2% decline in CHD deaths and a 6% increase in stroke deaths.^12^

### Statistical analyses

For each country, year and sex, we calculated the CHD and stroke mortality rates per 100 000 individuals, overall and in 5-year age groups ranging from 20–24 to 85–89 years. From these rates, we obtained the men-to-women ratio of CHD and stroke mortality rates. Bar charts were used to describe the sex-specific CHD and stroke mortality rate, by year; ranking countries by their overall CHD and stroke mortality rate. Line charts were used to characterise the sex-specific and age-specific CHD and stroke mortality rates, for each country and year. To examine whether the effect of ageing was the same over time in both sexes, we fitted separate linear regression lines for each country and sex, estimating the CHD and stroke rates by calendar year (coded 0, 1, 2, 3 for 1980, 1990, 2000 and 2010, respectively), 5-year age group (coded 0–13 for age groups 20–24 to 85–89, respectively), and their interaction. The interaction coefficients for women and men (representing the change in the effect of ageing on CHD and stroke mortality over time) were then plotted against each other, within country, to identify countries where such changes differed by sex. A series of boxplots was produced to examine the distribution, across countries, of men-to-women CHD and stroke mortality rate ratios, by study year and age (excluding the 80–84 and 85–89 year age groups due to the limited number of countries providing mortality rates in these age groups). Similarly, to evaluate the relative contribution of mortality from CHD and stroke, we made age-specific boxplots of the distribution of CHD-to-stroke mortality rates across countries, by sex. All analyses were performed in R V.3.1.1.

## Results

Of the world's 50 most populous countries, population size and mortality data were available for 26 countries for at least 1 year from 1980: 19 had data for 1980, 22 for 1990, 15 for 2000 and 15 for 2010. Malaysia, Peru, South Africa and Turkey each had 1 year of data; Argentina, Brazil, China, Colombia, Mexico, Philippines, Uzbekistan and Venezuela had 2 years; Canada, Egypt, Germany, South Korea, Thailand and Ukraine had 3 years; and France, Italy, Japan, Poland, Russia, Spain, UK and USA had 4 years (see online supplementary eTable 1).

**Figure 1:**
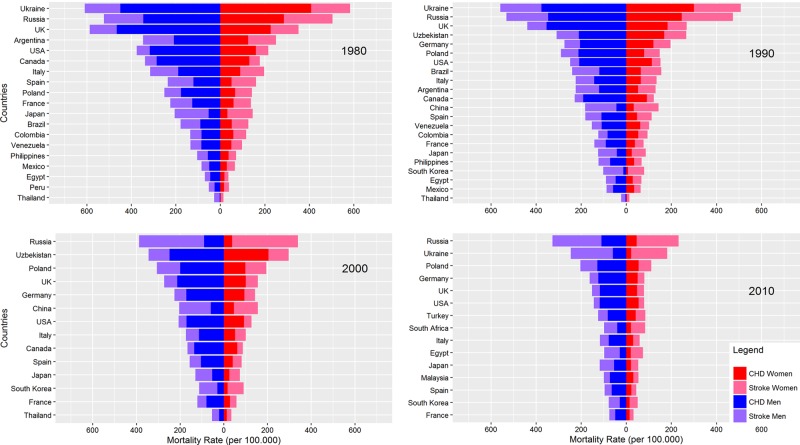
Mortality from CHD and stroke in women and men, by country and year. For aesthetic reasons, mortality rates for Ukraine in 2000 are not included in the Figure. In 2000, CHD mortality rates in Ukraine were 502 per 100 000 for women and 719 per 100 000 for men. For stroke, this was 221 per 100 000 for women and 412 per 100 000 for men.

Figure 1 shows the CHD and stroke mortality rates by sex, country and year for all available data. CHD mortality was higher than stroke mortality in men and women, except for some Asian countries. With few exceptions, mortality rates of CHD and stroke were considerably lower in 2000 and 2010 than in 1980 and 1990 in both sexes. The ranking of countries according to combined CHD and stroke mortality rates was broadly similar between men and women. In 1980, Ukraine, UK and Russia had the three highest combined CHD and stroke mortality rates for both sexes. In 2010, the corresponding top three countries were Russia, Ukraine and Poland.

**Figure 2:**
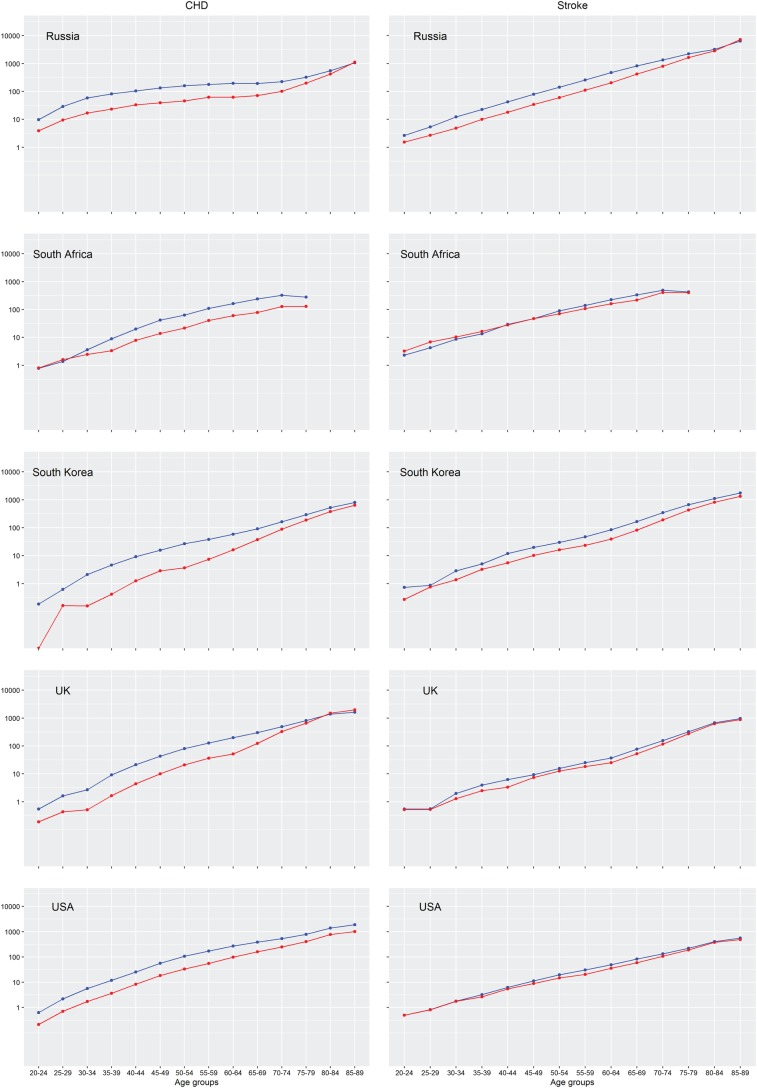
Age-specific mortality rates from CHD and stroke in women and men in 2010 in selected countries. Mortality rates are per 100 000 women (in red) or men (in blue) in each age group. Mortality rates for all countries and years are provided in efigures 1 and 2. CHD, coronary heart disease.

The 2010 CHD and stroke mortality rates by age and sex in five selected countries are shown in figure 2, and for all available countries and years in efigures 1 and 2. CHD and stroke mortality rates increased with age in men and women in all countries and years, with no apparent acceleration of mortality rates between particular age groups. For instance, there was no evidence of an effect of the female menopause. There was, however, variation between countries in the rate of increase in CHD mortality with ageing. For example, the increase in CHD mortality by age in Egypt, Russia and Ukraine was small compared with Canada, China, Germany, Italy, Poland, UK and USA. The rates of increase with ageing of the stroke mortality rates, in contrast, were similar between countries, and stoke mortality rates, by age, were much more similar between the sexes than were the CHD rates.

**Figure 3:**
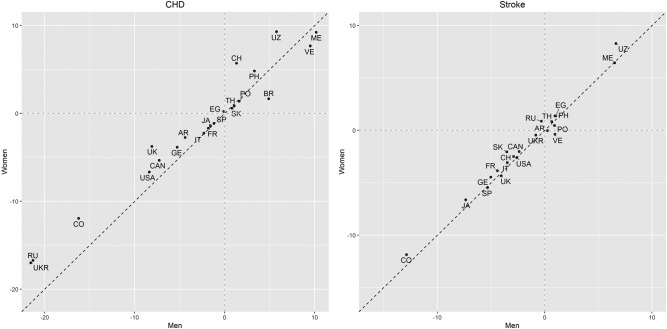
Estimated increment, over a 10-year period, in the additional rate of CHD and stroke mortality (per 100 000) for every 5 years higher age. For example, in Canada, it is estimated that, for a man at any age between 20 and 89 years, his increased chance of death from CHD within the next 12 months, compared with a male compatriot 5 years younger, has decreased by about 7 per 100 000 over any 10-year calendar period within the range 1980–2010 (for instance between 2000 and 2010). For the equivalent woman, the decrease was about 5 per 100 000. For stroke, in both sexes the decrease was about 2 per 100 000 in Canada. Estimates and SEs are provided in eTable 3. Country codes: ARgentina, BRazil, CANada, CHina, COlombia, EGypt, FRance, GErmany, JApan, ITaly, MAlaysia, MExico, PEru, PHilippines, POland, RUssia, South Africa, South Korea, SPain, THailand, TUrkey, UK, UKRaine, USA, UZbekistan, VEnezuela. CHD, coronary heart disease.

The effect of ageing on CHD mortality decreased with calendar time in most countries, notable exceptions being Mexico, Venezuela and Uzbekistan (figure 3 and see online supplementary eTable 3). This attenuation with calendar time was almost always greater in men than women, although most differences were small: China, Russia, Ukraine, UK and Uzbekistan had the greatest sex differential, always showing less of a positive effect in women, although Russia and Ukraine still had the greatest declines in the effect of ageing for women across all countries analysed. The corresponding results for stroke mortality were generally similar, although unlike for CHD, Russia and Ukraine showed virtually no change in the effect of ageing over the last 30 years on stroke mortality. Moreover, the attenuation of the effect of ageing on stroke mortality was similar between men and women.

**Figure 4:**
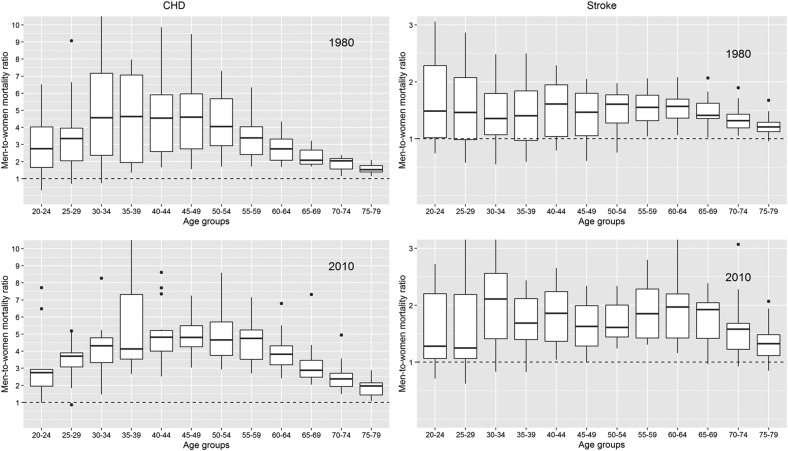
Men-to-women mortality rate ratios for CHD and stroke across countries in 1980 and 2010, by age. The band inside the box is the median mortality rate ratio, the bottom and top of the box are the first and third quartiles, the ends of the whiskers are placed 1.5 IQR distant from the lower and upper quartile. The dots represent observations outside that range. Countries contributing data to each of the years are listed in eTable 1. CHD, coronary heart disease.

The distribution of men-to-women CHD and stroke mortality rate ratios across countries is shown in figure 4. CHD mortality rates were consistently higher in men than in women, but, the magnitude of the ratio varied by age. For example, in 2010, CHD mortality was, on average, about four times higher in men than in women aged 30–60 years and the ratio declined gradually to two times higher rates at ages 75–80 years. Stroke mortality rates were more constant with age, yet were about 1.5–2 times higher for men than for women up until age 70 years and older where the ratio was closer to unity.

**Figure 5:**
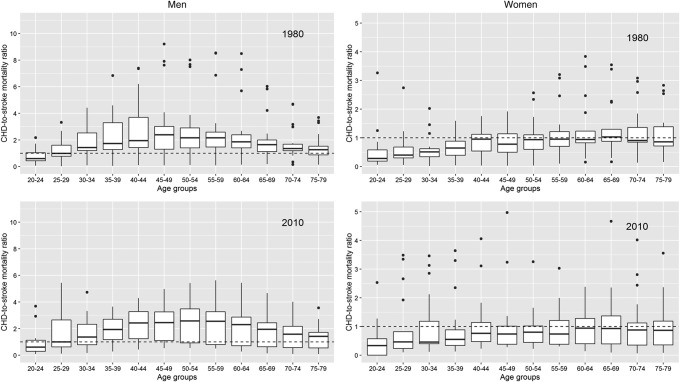
CHD-to-stroke mortality rate ratios for men and women across countries in 1980 and 2010, by age. Conventions as in figure 4. CHD, coronary heart disease.

The CHD-to-stroke mortality rate ratio differed between the sexes at all ages (figure 5). In men aged 40–64 years, CHD mortality was about twice as high as stroke mortality. The CHD-to-stroke mortality ratios were smaller at younger and older age, yet exceeded one in virtually all countries and years. In women, stroke mortality exceeded CHD mortality at almost all ages and in all years. However, ratios came closer to, or crossed, unity in the older age groups in all years except 2010.

## Discussion

Using WHO mortality and population data from 26 of the most populous countries worldwide, we demonstrated that CHD and stroke mortality rates have decreased substantially between 1980 and 2010 in men and women. On average, CHD mortality was up to five times higher in men than women, with some indication that CHD mortality at young and middle age had declined to a greater extent in men than women. Sex differences in mortality from stroke are smaller, yet rates remained approximately twice as high in men as compared with women up until late in life.

The declines in age-standardised CHD and stroke mortality rates in the present study are in agreement with previously reported trends seen in men and women in most parts of the world. The Global Burden of Disease study estimated that the global age-standardised CHD and stroke mortality rates dropped by 22% between 1990 and 2013.^1^ These achievements, however, are not shared by everyone, and global patterns may mask important differences in mortality trends between countries, age groups and sexes. For instance, unlike the successful reductions in CHD mortality rates in high-income countries, age-standardised mortality rates rose or stagnated in many other parts of the world, including Eastern Europe and countries in Central, South and East Asia. Within the European Union, the very modest reductions in mortality from CHD and stroke in the Eastern European member states have resulted in a widening of the East–West disparity.^13^ Also, although the rate of decrease in CHD mortality has remained stable or accelerated in most European countries in both sexes, there are some countries, including the UK, where the fall in CHD mortality rates has slowed down and might have plateaued in the past decade.^14^ Moreover, reports from the USA, UK, Canada and Australia suggest that the overall dramatic fall in CHD mortality is driven by large and continuing reductions among older men and women and conceal the apparent stagnation in younger adults, particularly young women.^15–18^ Under-representation of younger adults—especially younger women—in clinical studies on the presentation, risk factors and outcomes of CVD and a worse risk profile with more comorbidities might contribute to these slow improvements. Also, although CVD risk awareness among women and physicians has increased considerably over the past decades, it may be that older women have benefited most from these efforts.^3^ ^4^ ^19^ Major reductions in smoking prevalence, particularly in men, might also explain the apparent greater postponement of CHD mortality to later life in men than women.

The sex differences in CHD and stroke mortality rates reported here are in keeping with previous studies that found higher mortality rates in men than in women throughout most of life.^6^ ^8^ However, these sex differences are strongly affected by age and conceal the fact that men and women have a similar lifetime risk of CVD. Moreover, due to women's higher life expectancy, the overall CVD mortality rates are higher in women than in men, particularly for stroke which generally manifests at older age.^5^ ^6^ Population ageing is likely to increase the excess number of CVD deaths in women even further in future decades.

The reasons underpinning sex differences in CHD and stroke rates are not fully understood. However, a protective effect of sex steroid hormones, particularly oestrogen, on CVD risk in women has often been suggested.^20^ Despite evidence from animal and observational studies, clinical trials on the effects of exogenous oestrogen on the risk of CHD and stroke in postmenopausal women did not show a beneficial effect on CVD risk and suggested that supplementation could even increase the risk of stroke among women with history of CVD.^21–23^ However, a secondary analysis of the Women's Health Initiative showed that oestrogen could protect against CHD but not stroke among women within 5 years of menopause.^24^ Although the present study is not able to assess the impact of sex hormones on CVD mortality rates in women, we did not observe an apparent acceleration of mortality rates with advancing age.

Sex differences in the presentation of CHD may also explain the apparent smaller improvements in postponing CHD to older age in women. Compared to men, women have less obstructive coronary artery disease and warning signs of CHD in women are often regarded as ‘atypical’ and tend to go ignored, unrecognised or misdiagnosed.^25^ ^26^ As a consequence, women are less likely than men to receive pharmacological treatment for CVD risk factors or to be referred for diagnostic and therapeutic procedures.^27–29^ Suboptimal access to healthcare services leads to delays in diagnosis and treatment and to worse prognosis and outcomes for women with CVD. Awareness of CVD risk in women has increased substantially over the past decade, in part because of public campaigns and the development and implementation of CVD prevention guidelines for women in industrialised countries.^3^ ^4^ ^30^ However, continued efforts in the identification and quantification of sex differences in CVD, and understanding of their underlying mechanisms, are needed to better facilitate the development and implementation of strategies to prevent and treat CVD more efficiently in women and men, and to debunk the notion that CVD is a man's disease.

The WHO mortality and population databases provide valuable information about cause-specific deaths separately by age group, sex, country and year, enabling large-scale analyses and comparisons such as those between men and women conducted in the present study. While the data are standardised and generally of high quality, some limitations of using internationally aggregated vital records need to be taken into consideration. First, the unavailability of data, primarily from low and middle income regions, limited our ability to assess sex-specific mortality rates and their ratios for many populous countries and reduced the generalisability of our findings. Few countries had data on the oldest age groups, which limited our ability to assess whether and at what age sex differences in mortality rates declined further or ultimately reversed to excess mortality in women. Second, in countries with available data, there is variability in the coverage and the quality of the vital statistics databases collected and reported to the WHO. While this may affect the direct comparisons in mortality rates between countries, it is less likely to explain the differences between men and women within the same country. Third, countries used several versions of ICD over time which could have had an impact on the changes in CHD and stroke mortality rates over time.^12^ ^31^ However, the implications of changes in ICD coding may be minimised by the use of these relatively broad disease categories. Moreover, changes in ICD codes are likely to have affected men and women equally, indicating that the comparisons between sexes in the same year remain valid. Finally, for determining the effects of ageing, which was part of our remit, one would ideally be using longitudinal data. Since these are not widely available on a national scale, the repeat cross-sectional analyses used here are the best possible for obtaining country estimates.

In conclusion, while CHD and stroke mortality rates declined considerably between 1980 and 2010 in both sexes, there was some indication for stronger age-specific reductions in CHD in men than women. Mortality from CHD and stroke remains higher among men than women until old age across a range of economically, socially and culturally diverse countries.

## References

[1] The Global Burden of Disease 2013 Mortality and Causes of Death Collaborators. Global, regional, and national age-sex specific all-cause and cause-specific mortality for 240 causes of death, 1990–2013: a systematic analysis for the Global Burden of Disease Study 2013. Lancet 2015;385:117–71. 10.1016/S0140-6736(14)61682-2 25530442 PMC4340604

[2] The Global Burden of Disease 2013 http://vizhub.healthdata.org/gbd-compare/#. (Accessed 18-08-2016).

[3] Mosca L, Barrett-Connor E, Wenger NK Sex/gender differences in cardiovascular disease prevention: what a difference a decade makes. Circulation 2011;124:2145–54. 10.1161/CIRCULATIONAHA.110.968792 22064958 PMC3362050

[4] Mosca L, Hammond G, Mochari-Greenberger H Fifteen-year trends in awareness of heart disease in women: results of a 2012 American Heart Association national survey. Circulation 2013;127:1254–63, e1–29 10.1161/CIR.0b013e318287cf2f 23429926 PMC3684065

[5] George J, Rapsomaniki E, Pujades-Rodriguez M How does cardiovascular disease first present in women and men? Incidence of 12 cardiovascular diseases in a contemporary cohort of 1,937,360 people. Circulation 2015;132:1320–8. 10.1161/CIRCULATIONAHA.114.013797 26330414 PMC4590518

[6] Leening MJ, Ferket BS, Steyerberg EW Sex differences in lifetime risk and first manifestation of cardiovascular disease: prospective population based cohort study. BMJ 2014;349:g5992 10.1136/bmj.g5992 25403476 PMC4233917

[7] Ali MK, Jaacks LM, Kowalski AJ Noncommunicable diseases: three decades of global data show a mixture of increases and decreases in mortality rates. Health Aff (Millwood) 2015;34:1444–55. 10.1377/hlthaff.2015.0570 26355045

[8] Mozaffarian D, Benjamin EJ, Go AS Heart disease and stroke statistics--2015 update: a report from the American Heart Association. Circulation 2015;131:e29–322. 10.1161/CIR.0000000000000152 25520374

[9] Zhang XH, Sasaki S, Kesteloot H Changes in the sex ratio of stroke mortality in the period of 1955 through 1990. Stroke 1995;26:1774–80. 10.1161/01.STR.26.10.1774 7570724

[10] Parikh NI Sex differences in the risk of cardiovascular disease. BMJ 2011;343:d5526 10.1136/bmj.d5526 21896609

[11] World Health Organization. WHO mortality database: World Health Organization 2015 http://www.who.int/healthinfo/statistics/mortality_rawdata/en/ (accessed 24 Jan 2017).

[12] Anderson RN, Minino AM, Hoyert DL Comparability of cause of death between ICD-9 and ICD-10: preliminary estimates. Natl Vital Stat Rep 2001;49:1–32. 11381674

[13] Hartley A, Marshall DC, Salciccioli JD Trends in mortality from ischemic heart disease and cerebrovascular disease in Europe: 1980 to 2009. Circulation 2016;133:1916–26. 10.1161/CIRCULATIONAHA.115.018931 27006480

[14] Nichols M, Townsend N, Scarborough P Trends in age-specific coronary heart disease mortality in the European Union over three decades: 1980–2009. Eur Heart J 2013;34:3017–27. 10.1093/eurheartj/eht159 23801825 PMC3796269

[15] Izadnegahdar M, Singer J, Lee MK Do younger women fare worse? Sex differences in acute myocardial infarction hospitalization and early mortality rates over ten years. J Womens Health 2014;23:10–17. 10.1089/jwh.2013.4507 24206026

[16] O'Flaherty M, Allender S, Taylor R The decline in coronary heart disease mortality is slowing in young adults (Australia 1976–2006): a time trend analysis. Int J Cardiol 2012;158:193–8. 10.1016/j.ijcard.2011.01.016 21288580

[17] O'Flaherty M, Ford E, Allender S Coronary heart disease trends in England and Wales from 1984 to 2004: concealed levelling of mortality rates among young adults. Heart 2008;94:178–81. 10.1136/hrt.2007.118323 17641070

[18] Wilmot KA, O'Flaherty M, Capewell S Coronary heart disease mortality declines in the United States from 1979 through 2011: evidence for stagnation in young adults, especially women. Circulation 2015;132:997–1002. 10.1161/CIRCULATIONAHA.115.015293 26302759 PMC4828724

[19] Mosca L, Linfante AH, Benjamin EJ National study of physician awareness and adherence to cardiovascular disease prevention guidelines. Circulation 2005;111:499–510. 10.1161/01.CIR.0000154568.43333.82 15687140

[20] Tunstall-Pedoe H Myth and paradox of coronary risk and the menopause. Lancet 1998;351:1425–7. 10.1016/S0140-6736(97)11321-6 9593428

[21] Rossouw JE, Anderson GL, Prentice RL Risks and benefits of estrogen plus progestin in healthy postmenopausal women: principal results From the Women's Health Initiative randomized controlled trial. JAMA 2002;288:321–33. 10.1001/jama.288.3.321 12117397

[22] Boardman HM, Hartley L, Eisinga A Hormone therapy for preventing cardiovascular disease in post-menopausal women. Cochrane Database Syst Rev 2015;(3):CD002229 10.1002/14651858.CD002229.pub4 25754617 PMC10183715

[23] Hulley S, Grady D, Bush T Randomized trial of estrogen plus progestin for secondary prevention of coronary heart disease in postmenopausal women. Heart and Estrogen/progestin Replacement Study (HERS) Research Group. JAMA 1998;280:605–13. 10.1001/jama.280.7.605 9718051

[24] Rossouw JE, Prentice RL, Manson JE Postmenopausal hormone therapy and risk of cardiovascular disease by age and years since menopause. JAMA 2007;297:1465–77. 10.1001/jama.297.13.1465 17405972

[25] Shaw LJ, Bugiardini R, Merz CN Women and ischemic heart disease: evolving knowledge. J Am Coll Cardiol 2009;54:1561–75. 10.1016/j.jacc.2009.04.098 19833255 PMC2789479

[26] Anderson RD, Pepine CJ Gender differences in the treatment for acute myocardial infarction: bias or biology? Circulation 2007;115:823–6. 10.1161/CIRCULATIONAHA.106.685859 17309930

[27] Cabana MD, Kim C Physician adherence to preventive cardiology guidelines for women. Womens Health Issues 2003;13:142–9. 10.1016/S1049-3867(03)00034-3 13678805

[28] Humphries KH, Pu A, Gao M Angina with “normal” coronary arteries: sex differences in outcomes. Am Heart J 2008;155:375–81. 10.1016/j.ahj.2007.10.019 18215611

[29] Manteuffel M, Williams S, Chen W Influence of patient sex and gender on medication use, adherence, and prescribing alignment with guidelines. J Womens Health (Larchmt) 2014;23:112–19. 10.1089/jwh.2012.3972 24206025

[30] Mosca L, Benjamin EJ, Berra K Effectiveness-based guidelines for the prevention of cardiovascular disease in women--2011 update: a guideline from the American Heart Association. Circulation 2011;123:1243–62. 10.1161/CIR.0b013e31820faaf8 21325087 PMC3182143

[31] Janssen F, Kunst AE ICD coding changes and discontinuities in trends in cause-specific mortality in six European countries, 1950-99. Bull World Health Organ 2004;82:904–13. doi:/S0042-96862004001200006 15654404 PMC2623106

