# Supplementary material for: Sex differences in coronary heart disease and stroke mortality: a global assessment of the effect of ageing between 1980 and 2010
Source: BMJ Glob Health. 2017 Mar 27;2(2):e000298. doi: 10.1136/bmjgh-2017-000298 (PMC5435266; doi:10.1136/bmjgh-2017-000298)
Supplement: supplementary eTables [file bmjgh-2017-000298supp_eTables.pdf]

Supplementary appendix

Sex differences in coronary heart disease and stroke mortality: a global assessment of the effect of ageing between 1980 and 2010

Sophie H. Bots, Sanne A.E. Peters, Mark Woodward

eTable 1: Overview of data available for the world's 50 most populous countries

Country	Population data available	Mortality data available	Years used in study
Afghanistan	NA	NA	
Algeria	NA	NA	
**Argentina**	1955-1996	1966-2012	1980, 1990
Bangladesh	NA	NA	
**Brazil**	1979-1995	1977-2012	1980, 1990
**Canada**	1950-2005	1950-2011	1980, 1990, 2000
**China**	1987-2000	1987-2000	1990, 2000
**Colombia**	1955-1994	1953-2011	1984, 1991
Congo.Dem.Rep	NA	NA	
**Egypt**	1955-2013	2001-2013	1980, 1991, 2010
Ethiopia	NA	NA	
**France**	1950-2012	1950-2011	1980, 1990, 2000, 2010
**Germany**	1990-2013	1990-2013	1990, 2000, 2010
Ghana	NA	NA	
India	NA	NA	
Indonesia	NA	NA	
Iran	1983/95/97	1974 ~ 1987	
Iraq	NA	2008	
**Italy**	1950-2012	1951-2012	1980, 1990, 2000, 2010
**Japan**	1950-2013	1950-2013	1980, 1990, 2000, 2010
Kenya	NA	NA	
Korea, North	NA	NA	
**Malaysia**	2007-2008	2000-2008	2008
**Mexico**	1958-1995	1955-2012	1980, 1990
Morocco	NA	2008-2012	
Mozabique	NA	NA	
Myanmar	NA	1977-1978	
Nepal	NA	NA	
Nigeria	NA	NA	
Pakistan	NA	1993-1994	
**Peru**	1958-1983	1966-2012	1980
**Philippines**	1950-1996;2005	1963~2003;2008	1981, 1994

**Poland**	1950-2013	1959-2013	1980, 1990, 2000, 2010
**Russian Federation**	1980-2011	1980-2011	1980, 1990, 2000, 2010
Saudi Arabia	NA	2009;2012	
**South Africa**	2007-2013	1993-2012	2010
**South Korea**	1985-2012	1985-2012	1990, 2000, 2010
**Spain**	1950-2013	1951-2013	1980, 1990, 2000, 2010
Sudan	NA	NA	
Tanzania	NA	NA	
**Thailand**	1950-1994;2002	1955-2006	1980, 1990, 2000
**Turkey**	2009-2013	2009-2013	2010
Uganda	NA	NA	
**UK**	1950-2011	1950-2013	1980, 1990, 2001, 2010
**Ukraine**	1981-2012	1981-2012	1990, 2000, 2010
**USA**	1950-2007	1950-2010	1980, 1990, 2000, 2010*
**Uzbekistan**	1981-2005	1981-2005	1990, 2000
**Venezuela**	1950-1994	1955-2009	1980, 1990
Vietnam	NA	NA	
Yemen	NA	NA	

Countries are alphabetically ordered. Countries in bold were included in the present study.

*Population data from 2007 was used

eTable 2 Countries with ICD coding practices that differed from common practice*

**Country**	**Year(s)**	**ICD code used**
China	1980, 1990	ICD 9th revision, Special List of causes as reported by China
Italy	2000	ICD 9th revision, Basic Tabulation List
The Philippines	1981	ICD 8th revision, List A
Russia	1980, 1990	ICD 9th revision, Special List of causes (condensed) as reported by some countries of the newly independent States of former USSR
Russia	2000, 2010	ICD 10 condensed Mortality Tabulation List
Ukraine	1990, 2000	ICD 9th revision, Special List of causes (condensed) as reported by some countries of the newly independent States of former USSR
Ukraine	2010	ICD 10 condensed Mortality Tabulation List
Uzbekistan	1990, 2000	ICD 9th revision, Special List of causes (condensed) as reported by some countries of the newly independent States of former USSR

*Common practice entails the use of ICD-9 in the years 1980 and 1990 and ICD-10 in the years 2000 and 2010.

eTable 3: Estimated increment, over a 10-year period, in the additional rate of CHD and stroke mortality (per 100,000) for every 5 years higher age.

	CHD				Stroke			
	Men		Women		Men		Women	
Country	Estimate	SE	Estimate	SE	Estimate	SE	Estimate	SE
CAN	-7,27	3,95	-5,35	3,61	-2,38	1,76	-2,02	1,85
EG	-0,08	0,19	0,21	0,18	1,02	0,33	1,37	0,37
FR	-1,81	0,65	-1,69	0,55	-4,41	1,16	-3,87	1,12
GE	-5,27	2,81	-3,86	2,33	-5,02	1,98	-4,47	2,00
IT	-2,31	1,52	-2,26	1,46	-3,48	1,84	-3,08	1,87
JA	-1,57	0,60	-1,39	0,49	-7,37	1,84	-6,63	1,66
PO	1,62	0,83	1,38	0,79	0,91	0,78	0,46	0,83
RU	-21,30	3,98	-16,72	3,76	-0,31	3,29	0,87	3,80
SK	0,82	0,56	0,59	0,50	-3,52	1,89	-2,06	1,52
SP	-1,19	0,75	-1,14	0,74	-5,32	1,64	-5,46	1,73
TH	1,06	0,18	0,84	0,18	0,66	0,18	0,83	0,23
UK	-8,09	1,73	-3,75	1,56	-4,05	1,26	-4,35	1,44
UKR	-21,53	6,11	-17,00	5,88	-0,84	2,00	-0,45	2,27
USA	-8,38	2,26	-6,67	2,02	-2,60	0,82	-2,61	0,89
AR	-4,39	5,54	-2,74	5,38	0,25	4,77	-0,04	5,17
BR	4,89	3,03	1,66	3,91	24,51	7,09	17,75	7,82
CH	1,32	2,46	5,71	2,83	-2,90	4,28	-2,51	3,72
ME	10,17	4,06	9,22	3,97	6,53	3,02	6,44	3,03
PH	3,31	4,54	4,82	5,18	0,96	2,56	1,38	2,62
UZ	5,76	18,63	9,29	18,15	6,64	6,14	8,28	7,34
CO	-16,23	6,58	-11,93	4,98	-12,88	4,81	-11,86	4,56
VE	9,51	5,90	7,67	6,16	0,95	3,68	-0,37	5,01

Country codes: **AR**gentina, **BR**azil, **CA**nada, **CH**ina, **CO**lombia, **EG**ypt, **FR**ance, **GE**rmany, **JA**pan, **IT**aly, **MA**laysia, **ME**xico, **PE**ru, **PH**ilippines, **PO**land, **RU**ssia, **S**outh **A**frica, **S**outh **K**orea, **SP**ain, **TH**ailand, **TU**rkey, **U**nited **K**ingdom, **UKR**aine, **U**nited **S**tates of **A**merica, **UZ**bekistan, **VE**nezuela
